# Conceptualizing an athlete support ecosystem for women in professional team sport

**DOI:** 10.3389/fspor.2026.1942522

**Published:** 2026-10-07

**Authors:** Tracy Taylor, Clare Hanlon, Wendy O'Brien

**Affiliations:** 1 College of Business and Law, RMIT University, Melbourne, VIC, Australia; 2 Institute for Health and Sport, Victoria University, Melbourne, VIC, Australia

**Keywords:** professional sport, sport organisations, women athletes, women's sport, women's sport participation

## Abstract

The expanse of women’s professional team sports has burgeoned in the last ten years. The inroads made by women’s leagues have raised questions about how to equitably and sustainably support, finance, and manage and foster the growth and development of women athletes. Women’s leagues have unique characteristics that warrant recognition and positioning which are different from their counterpart men’s leagues and enable gender specific value creation. However, the development of many women’s leagues, both stand alone and those that share league governing bodies with the men’s leagues, have positioned women in precarious and unsustainable employment condition with little consideration of gender relevant work conditions, culture and safety, career pathways and representation. We argue that an alternative framework for women’s leagues, that attends to the gendered needs of women athletes, can foster equitable approaches that support sustainable practice and better employment outcomes. In this paper we propose a gendered conceptual framework, termed a Women Athlete Support Ecosystem, as a holistic way to enhance professional team athlete support and development for women. Drawing on literature and framework analysis we re-frame a socio-ecological model to consider a feminist intersectional approach proposing four key areas: individual and sustainable care; interdependence of athlete wellbeing; organizational practices; and community connection. Our approach to athlete support and development incorporates sport as community, and concepts embodied in sustainability, flourishing, wellbeing, and care.

## Introduction

In the past ten years there has been an unprecedented global growth of Women's Professional Teams Sports Leagues (WPTSL) (1–8). Many leagues are routinely setting new attendance records, breaking new ground, reshaping perceptions about women's sport, gaining new sets of fans, and creating a different way of positioning women's sport (9). Women's leagues operate across traditionally women-dominated sports (e.g., netball) and traditionally male-dominated sports (e.g., cricket, football and rugby codes), and in the latter can face inherent hegemonic masculine cultures (7). Questions have been raised about the veracity of transposing traditional performance success indicators of men's professional sport leagues (e.g., revenue generated, sponsor investment, spectator attendance, etc.), to measure the success of women's leagues (6). While useful, these indicators primarily focus on hyper-commercialized and masculine models of success linked to economic growth and return on investment (9, 10), it limits the potential to identify and value the unique characteristics of women's leagues. Further, Alcântara et al. (11), suggest that professionalization cannot be viewed solely as competitive success and/or commercial growth but needs to fully consider the labour and developmental conditions of women athletes.

Over the past few decades several WPTSL have ceased operations due to financial issues (12) or have not lived up to expectations of growth (13). This has been attributed in part to the negligible level of initial start-up investment in these women's leagues. These leagues have grappled with a chronic lack of infrastructure, limited revenue sources and minimal talent development pathways from community through to elite levels, all which add to the challenge of profitability and success (14). Public commentary often disparages these leagues for being cross subsidized by their national governing body/league owners or under-written by the corresponding men's team. While linking WPTSL to a “master brand”, that is an established men's competition, may provide some benefits; organizations with “integrated” women's teams also need to value and recognize the distinctive characteristics of these teams (15, p. 23). These tensions raise questions about the sustainability of women's leagues and the “preconceived biased notion in the industry that women professional sports are very unlikely to be successful” (12, p. 114). Merely emulating a gendered male professional sport model can result in women being inherently “othered” (9) or judged on criteria that is inappropriate or ill-suited to the women's league. Alcântara et al. (11, p. 3), point to an absence of an integrated approach that considers “institutional structures, organizational practices and athletes' careers within a single interpretive frame.” We argue, it is time to conceptualize a more sustainable, person centered, community-oriented approach that is framed within feminist ethics, flourishing and celebrates the unique elements of women's leagues. As O'Brien et al. (16) suggest, WPTSLs offer an opportunity to operate differently and care for women athletes in ways that are gender appropriate.

Within this rapidly changing milieu, our research aims to develop a conceptual framework directed at a gendered approach to professional team athlete support and development. We develop an alternative to growth-based professionalization through re-framing an ecological-intersectional approach (17) to incorporate feminist sustainability, care, flourishing and wellbeing. Our thinking is informed by notions of intersectionality (17) and a sport as community orientation (18). This provides us with a foundational basis to encourage conversations that embrace complexity, uniqueness, equity and long-term sustainability. The conceptual framework was informed by our multi-year research project with WPSTLs and is presented in the following section. The methodology is then outlined, drawing on a conceptual framework approach aligned with framework analysis.

## Conceptual framework

We extend Taylor et al.'s (19), research examining the multi-layered barriers and supports in women's semi-professional rugby league in Australia, through an ecological-intersectional framework. Drawing on this work, we propose a Women Athlete Support Ecosystem (WASE) focused on individual sustainable care, interdependence of athlete wellbeing, organizational culture and community connection. Viewing WPTSL through an ecosystem lens, allows us to focus on the interconnected domains through which women's professional sport is produced, through bidirectional relationships, that both shape and is shaped by the other domains. An ecosystem approach emphasizes interdependencies and the co-creation of value across multiple actors and contexts (20, 21); and enhances our ability to analyze, plan and potentially transform women's professional sport in holistic, equitable and sustainable ways. The domains are in turn guided by the underlying principles of sustainability, equity, intersectionality and care. Feminist flourishing, wellbeing and inclusion are positioned as outcomes, made possible through the interacting elements of the ecosystem.

Similarly, the Ecological-Intersectional Model (17) recognizes intersectionality in the individual layer of the model, but not across all levels. However, LaVoi (17) importantly argues that an intersectional approach, allows an examination of how individual experiences intersect through “systemic injustices, oppression, and social inequality” (p. 17). We extend on this to propose that intersectionality is a principle that operates across all domains of the framework. Addressing women's gendered needs is often narrow, focused on performance aspects such as the impact of menstrual cycle rather than considering broader socio-cultural factors that may affect wellbeing (22). LaVoi (17) highlights gender, along with age, parental status, sexual identity, and ethnicity as key identities that require attention to negate oppression, inequities, inequalities and injustices. Steel et al. (23) also argue intersectionality should be central to any understanding of women athletes' experiences, as intersecting systems of power “(re)produce inequities … including but not limited to … race … sexuality and ableism.” (p. 2688). Gender is not produced independently of these identities (23), the latter intersect to shape how athletes experience organizational culture and care in ways that can enhance or diminish their flourishing, wellbeing and inclusion. Organizational environments may produce different opportunities, depending on an athlete's intersecting social and material circumstance. Turelli et al. (24) propose that marginalized communities can exercise voice and agency, drawing on lived experience, and communal support, to pursue advocacy strategies that potentially transform sports organizations. An intersectional approach recognizes that supporting athletes requires flexible systems that are responsive to diversity and avoid treating women as a homogenous category. Our WASE concept is intended as a guide to discuss the conditions that enable women with differing intersecting identities to be supported and flourish.

Including intersectionality requires attention to how power is exercised across the domains to shape athlete's experiences. LaVoi (17) incorporates a Foucauldian interrogation of how power is exercised, from not only top down, but also how individuals may create change through resistance or disruption (bottom up). This approach allows us to think about countering and challenging dominant masculine cultures within the sports ecosystem. How power is exercised, particularly within gendered organizations can also impact wellbeing, development and performance (17). Interrogating masculine power structures and gender ideologies is central to creating opportunities for change (17). This approach raises the following questions: do women have the resources necessary to sustain careers as professional athletes? Are these women included in decision making about their working conditions? Are their experiences valued? Are their needs visible?

While women may resist and disrupt power structures from the bottom up, responsibility should not be placed on them to produce organizational change. Rather, women should be supported to exercise agency to participate in decision making, and contest discriminatory practices through appropriate reporting mechanisms and protections. WASE challenges conventional professional sporting models which ask women to self-adapt to a sporting system historically framed around masculine norms. We invert this to propose that sports organizations adapt their employment arrangements to attend to women's gendered needs. Similarly, O'Brien et al. (25) argued organizations should change their practices to include women, rather than women attempting to change to fit into the organization.

Our conceptual approach has been to create a framework focused on practices that attend to the realities of women's everyday lives. As such our ecosystem is framed around care as a principle of organizational support. Organizational care has a focus on three key values, affirmation, empowerment and belonging (16), actioned through practices that allow women to feel valued, and treated as whole people, not just athletes, who have diverse needs, identities and circumstances. Athletes are empowered to act through supportive practices and have agency and resources to influence their sporting environment. Equitable relations with organizations, teammates, and community create connection and inclusion (16). Care therefore seeks to promote flourishing, wellbeing and inclusion through transforming organizational values into a holistic, supportive and equitable environment.

Challenging and resisting power relations and values, often connected to commercialization and external markets in the professional sports domain, is a central focus of the proposed framework and offers a critique of “market-based” exchanges (26, p. 233). Mellor (27) argues that the commodified market system imposes boundaries around what is valued and what is externalized, with “patterns of exclusion” (p. 147) operating along gendered lines, treating people as assets to be exploited for return on investment. This framing provides another way to critique masculine and other discourses of exclusion that do not account for differences within conventional sport professionalization models. Thus, we link the proposed framework to a feminist economic understanding of sustainability which combines:

conditions of existence where human beings achieve their potential without exploiting the labor and resources of other individuals, groups or societies with a level of ecological replenishment necessary not only for human futurity but for the continued existence of other species and their ecosystems (28, p. 1).

Sustainability is a underlying organizing condition, recognizing support as ongoing, reciprocal and capable of being maintained over time. Sustainability is therefore not only about ensuring that people are not treated as commodities, but as social and relational beings with differing needs. This concentrates our attention on sport as community, in contrast to sport as commodity which is largely instrumental, focused on control, normative growth and management of athletes, and is potentially unsustainable (29). In a sport as community approach, organizations can invest in an athlete's self-determination, learning and personal growth, while also recognizing communal relations (29). Dohlsten et al. (19) argue that this approach to sustainable athlete development “is characterised by a focus on process [and] athletes as human beings” (p. 729). This suggests the incorporation and co-creation of knowledge and negotiated processes of social justice. For athletes, sustainable solutions would focus on context specific individual needs rather than a one-size-fits-all approach (29). Athletes also can shift their thinking from a commodity focus, towards one that is more sustainable, to avoid risks to their health and well-being (19). The processes of sustainability create the conditions for inclusion, wellbeing and flourishing. While LaVoi (17) does not directly address sustainability, the interacting layers offer the possibility to broaden the social-relational aspect, and the interrogation of power to challenge unequal and unsustainable models of growth. LaVoi's (17) work provides the opportunity to re-examine the socio-cultural context of sports organizations that re-create inequalities and practices to create different supports for those within the system.

LaVoi (17) importantly includes a development trajectory which occurs over time and is impacted by the various layers. We extend on LaVoi's work (17) to argue that the layers of influence are bi-directional. The presence or absence of support in one domain can flow across the ecosystem, undermining its sustainability or acting to transform practices. For example, while organizational culture can affect athletes' wellbeing;athletes' advocacy and collective action through players' unions can also influence organizational practices. Sports organizations can implement policies and practices that demonstrate women's equality and hence challenge or transform ingrained social norms of gender inequality in the workplace. WASE considers the supports needed across the ecosystem to ensure women can flourish and be supported in feminist, sustainable and equitable ways. WASE proposes that athlete support is produced through the interactions of all domains, rather than the outcome of any single intervention. How WASE expands on LaVoi's Ecological-Intersectional Model, is outlined in Table 1.

**Table 1:** Alignment and expansion of the ecological-intersectional model using the WASE framework.

Ecological-Intersectional Model	WASE Framework
Examines multiple levels of influence	Emphasizes a bidirectional, relational and interdependent ecosystem. Focuses on how the relationship between the levels can be reconfigured to include women.
Intersectionality focuses on the individual level, identifying how multiple identities and systems of oppression interact.	Embeds intersectionality, and sustainability, equity and care as key principles that operate across all levels of the framework. This also challenges masculine norms towards gender-responsive and intersectional practices.
Power is exercised from top-down and bottom-up through resistance and disruption.	Interrogates the patterns of exclusion, and dominant masculine cultures asking whether these structures are appropriate and sustainable for women's professional sport. Re-frames these patterns to guide organizational practices to value women. Women's agency is emphasized and supported throughout the ecosystem.
Wellbeing and flourishing are not specific features, but the multiple layers of influence provide a useful starting point to identify the bidirectional flow.	Wellbeing and flourishing are conceptualized as outcomes produced relationally and structurally across all layers of the framework. Connects wellbeing and flourishing to agency, power, and reconfigured organizational practices.
Positions the athlete at the heart of the system.	Embraces this approach but shifts attention from the athlete to the ecosystem of actors and relations through which women's professional sport is produced. Also incorporates sport as community, emphasizing athletes as human, relational and social beings, rather than performance orientated commodities.
Incorporates a development trajectory in which change occurs over time.	This is an important element that WASE builds on to emphasize that athlete development is not necessarily linear, allowing for injury, motherhood, etc.
Tends to focus on barriers, supports, experiences or outcomes.	Has a transformative agenda, focusing on what a sustainable and socially just women's professional ecosystem would incorporate. This is a shift from athlete adaption to ecosystem or organizational adaption.

Links between equity and flourishing and wellbeing are also considered in WASE. A feminist flourishing in professional women's sport is not about being the same as men's sport, it is about equity and social justice (30). Thus, there is a need to rethink how women who play professional sport can thrive as athletes, workers, leaders, carers, and humans, with dignity, power, and joy. Feminist flourishing considers how embodied capacities can be enhanced or diminished by the power relations in which people are situated (30). Flourishing can occur in sports context that foster equity and diversity, athlete health and wellbeing require a holistic, multidimensional and gender responsive approach that fosters an inclusive, equitable environment (31). Feminist flourishing situates empowerment and agency within broader socio-political power dynamics (31). Within this framing, wellbeing is not an individual outcome to be optimized or measured through economic metrics instead, viewed as produced through renegotiated structural arrangements, power relations, and collective agency. As Thorpe et al. further argue, without an approach that acknowledges gender, “policy and practice will continue to overlook the crucial relations of power that can erode wellbeing or enhance capacities for collective and individual support” (p. 8). WASE incorporates inclusion and wellbeing in the present and flourishing over time, by aligning ecological sustainability with equity, caring, and power-aware sporting systems.

## Methods

Our conceptual framework evolved through discussions about how to develop an approach to women's professional sport that is gender-responsive and grounded in principles of equity, sustainability, intersectionality and care. Our goal was not to conduct a systematic or scoping review of literature, rather to draw on an ecological-intersectional model (17, 32) as a starting point to focus our thinking regarding organizational approaches to women's professional sport. It is important to note that a conceptual framework differs from a theoretical framework, in that it is constructed by researchers; the researchers draw on ideas from the literature, to engage in “thought experiments … [and develop] emergent ideas” (33, p. 7). The ecological-intersectional model provided the opportunity to consider relational connections between the elements of our framework, describe the relationships between these elements and draw on a focused engagement with key literature, adding to the rigor of the research design (33). The ecological-intersectional model and a feminist collaboration provided the tools to begin illuminating the relationships between the elements. This approach allowed us to consider issues such as athlete wellbeing, care, organisational culture, intersectionality, sustainability and community that we thought needed to be included in any model for women's professional sport. These areas also then guided our targeted literature searches.

While there is no single method defining how to develop a conceptual framework (33), framework analysis allowed us to create an analytic framework and then apply the framework to existing literature (34). Goldsmith (34) argues that “[r]esearchers can also use framework analysis to push beyond a thematic description of a phenomenon to the development of multi-dimensional typologies or theory development” (p. 2062). Rather than follow the step-by-step process, we adopted these steps as a guide, embracing the provisional aspect of the framework that allowed refinement across various stages.

As feminist sports scholars, we were familiar with much of the literature related to WPTSL. Familiarization is the first step in developing the categories for a framework (35). The body of literature we engaged with pertained to athlete wellbeing and care. As we considered this literature we continued to move through iterations of our conceptual framework. Parkinson et al. (35) argue that framework analysis “emphasizes how both *a priori* issues and emergent data driven themes should guide the development of the analytic framework” (p. 112). This approach enabled us to build a feminist, literature-informed analytical framework that is critically grounded, and reflexive. In essence, we drew on our framework to create a dialogue with extant literature and feminist theory.

As we applied the analytic framework to literature, we identified supportive evidence and developed the framework further. The ecological-intersectional model enabled the mapping of the literature onto individual, interpersonal, organizational, community and policy-level factors. We refined our framework as new ideas developed, moving between framework development and reading through literature (35). This is similar to “indexing” (34), as we explored the categories of the WASE framework by conducting searches with key words to find literature related to the four levels of the framework. At this stage we narrowed our literature to focus specifically on key areas, with searches conducted around target words such as coaching, contracts, family, intersectionality, salaries and sexuality. We wanted to dig deeper and create another set of layers to the conceptual framework associated with a gendered approach, and which incorporated feminist flourishing, wellbeing and care. This process was “an exercise in intellectual organization and clarity that often considers what is learned, known, and experienced” (33, p. 7). Charting or ordering the literature gave us the opportunity to further refine the framework. It was at this stage we began to develop a visualization that captured the interacting elements of the WASE Framework.

In the final stage of mapping and interpretation, we drew our insights together to refine our WASE Framework. We were mindful to be creative and follow our analysis to where it might take us. For example, we used the analysis to continually inform our conceptual approach, and this approach also informed the analysis. We worked through various drafts, discussing possible ways forward. As feminists, we also acknowledge our positionality, we are cis-gender, white sports scholars embedded in various power relations, with multiple and shifting identities, and this influences the standpoint from which we produce knowledge. In turn, this led us to constantly consider intersectionality in the conceptual framework, questioning our assumptions and reflecting on moments of discomfort or bias (36). Hence our collaboration was an ongoing dialogue, reflecting, questioning, and taking time to allow ideas to develop (37).

## Building the proposed WASE framework

The three-step conceptual approach identified elements within the WASE Framework to guide WPTSLs on practices that support athlete development and wellbeing. We recognize these elements are a start, a benchmark to build upon within each WASE layer. Individually, these layers interact bidirectionally, to highlight how personal, relational, institutional and societal dimensions form the foundation for associated elements focused on flourishing, wellbeing and care (Figure 1). This conceptualizes the relationship between the four domains as interdependent elements, through which women's professional sport can be re-thought and produced. This allows us to go beyond normative recommendations, evidence-based propositions, and adjacent evidence, these elements work in conjunction with our conceptual framework. Thus we avoid being overly prescriptive and engage critically with extant literature to inform the framing of the model by examining how these elements may interact in an ecosystem. At this conceptual stage, WASE is a framework, ready to be empirically tested and to guide future research.

**Figure 1:**
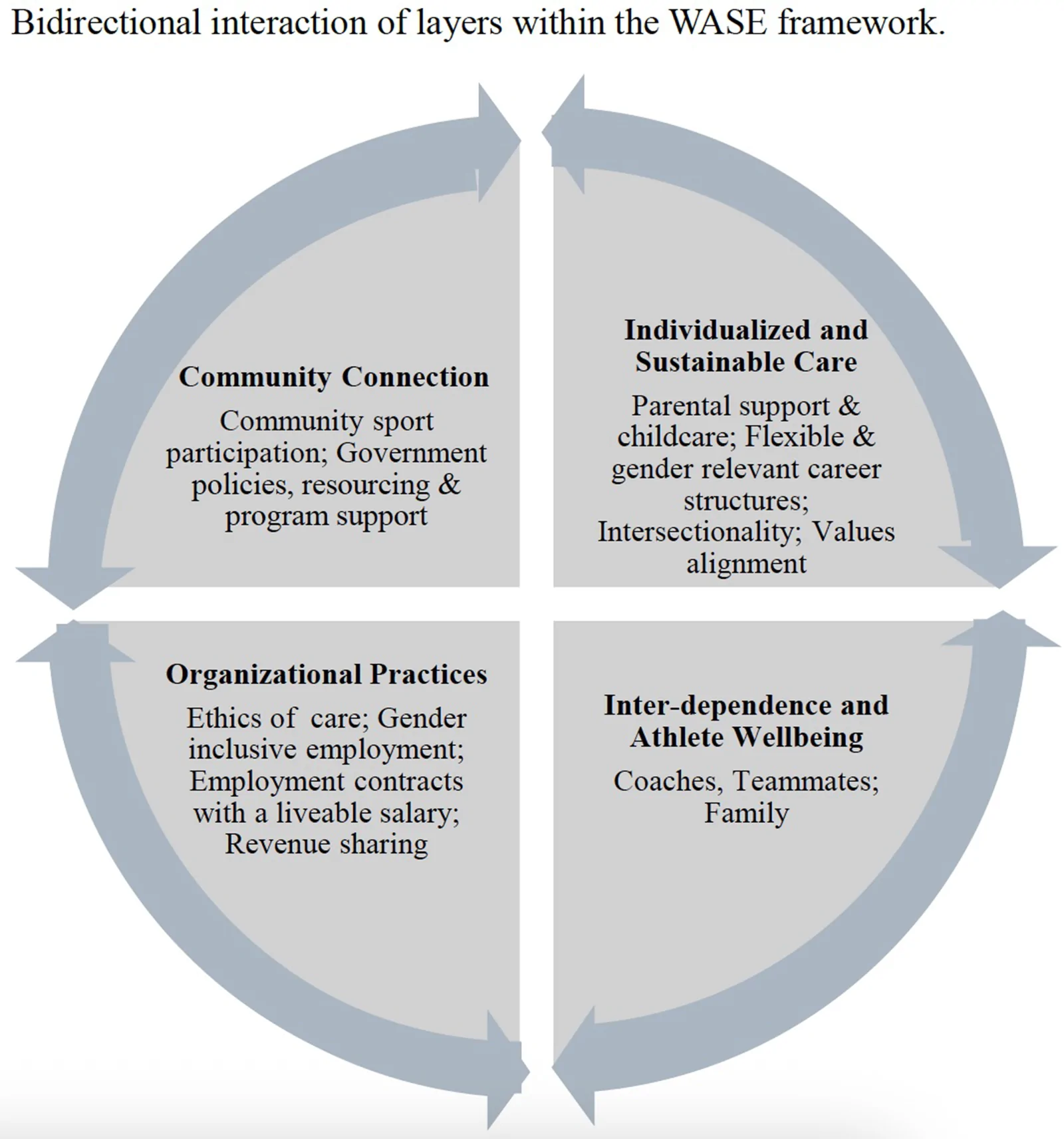
Bidirectional interaction of layers within the WASE framework.

### Individualized and sustainable care

At the individual level, the complex interplay of intersectional elements is central to the WASE framework. The framework provides for identifying, respecting and supporting women's diverse identities. Women's experiences in professional sport are not homogenous, and they can be similar and different to other women. Given the extant literature on WPTSL, including a lack of consideration around motherhood (38), there is a need to recognize individual family circumstances. This includes: (i) care provider roles, (ii) flexible and gender-relevant career structures; (iii) intersectionality; and (iv) values alignment.

Recognising the *care provider roles* of women encompasses provision for pregnancy and parental care leave, time off to recover after the birth of a child (16). Research (e.g., 3) indicates that family and women's caring roles are a barrier that women athletes are constantly negotiating to attain their career aspirations. Athletes' access to child-care provisions would enable women to return to sport in a way that supports their changing circumstances. As Barker-Ruchti et al. (19) argue, this may require an approach adapted to athlete's specific situations.

*Flexible and gender relevant career structures* for athletes, especially in semi-professional leagues where athletes may have other jobs or study, allows for a sustainable workload balance, with long-term development prioritized over short-term gain. Dual-career options for athletes have been found to produce significant benefits (e.g., resilience; post-athlete work related skill development), associated with having another career outside of sport; although research on the buffering effects of a dual career is still nascent (39). Nevertheless, employment considerations for allowances for study and work, with attention to women's multiple caring demands, and post-career development programs, can assist athletes to exit their playing career successfully (3). The provision of non-linear and individualized talent requires innovative approaches (40) pertinent to WPTSL. This may include normalization of flexible and individual career structures with allowance for career breaks and return to play guarantees to support women's career transitions. The development of these flexible pathways encourages sustainability, underpins the professional leagues and future proofs these competitions (5). Women could then negotiate their own career structure arrangements based on issues that specifically address their needs through gender-balanced player unions/representative bodies to enable women athletes to be meaningfully represented in decision-making processes. Women's gendered considerations should not be obscured by prevailing masculine power relations.

The third aspect recognizes *intersectionality* considerations for women athletes in professional sport. Ethnicity, indigeneity and an individual's cultural identities need to be foregrounded, including respect for cultural obligations, faith-based commitments, ethnic and gender diversity, language, socio-economic status and disability (41). This involves moving beyond single attribute initiatives to address these complex layers and bringing flexible work, tailored benefits, and inclusive safety protocols into the workplace. As women experience the workplace differently, changes mean going beyond single or generic policies, to consider how issues, such as insecure employment and gender intersect to affect wellbeing and flourishing. Confidential disclosure pathways enable women athletes to express their needs or report harassment or discrimination without fear of reprisal.

Celebrating and allowing for identities to be expressed and recognized is important to allow athletes to feel valued within an organization (31). Embracing multiple intersecting identities is a key aspect of creating an ethics of care that recognizes individual diversity, enabling athletes to reach their potential. While cultivating a culture of belonging may be constrained by organizational practices (16), we contend that creating inclusive practices that attend to intersectionality could help retain women players within the sport. Talent would then be retained, competitive continuity built, and leagues becoming more stable, contributing to longer term sustainability. In addition, a sense of belonging, and valuing of intersectional identities contribute to athlete wellbeing (41, 42).

Culture and *values alignment* are important in the clubs, women athletes choose to play for (19). Women athletes are more likely to invest in positive ways in “institutions that reflect values of care and equality, without which they can feel excluded” (17, p. 9). Social and psychological factors, such as gendered expectations, sexism and inequalities in the way women athletes are treated, also play crucial roles in their wellbeing (22). Hence, leading into the opportunity for organizations to recognize and enact their values within a WASE framework. This is not a focus on *fixing* women, but re-framing organizational practices towards an athlete-centered approach. Given the prevalence of gender discrimination and harassment in professional sport (43), reforms are clearly needed to provide athletes with a platform to speak up about practices that may threaten their wellbeing. Meaningful organizational accountability can ensure that athlete wellbeing takes precedence over commercial and performance priorities. Empowering women to have agency within an organization, exercise control over their lives, and have a voice to challenge discrimination and violence, can assist with negating gender discrimination and abuse (44). This may be achieved through measures such as anonymous reporting, independent welfare officers, and protection from reprisals. In turn, this could give women control over their lives more broadly (45). As Streetman and Heinrich (45) note, empowerment “helps defy gender stereotypes, boost self-esteem, encourage inclusion, and promote leadership among women and girls through participation in sports” (p. 815).

### Interdependencies and athlete wellbeing

Interpersonal relationships play a critical role in an athlete's support system (32). Three primary “roles” are evidenced to provide critical relational and cultural support to athletes: (i) coaches; (ii) teammates and (iii) family. Taylor et al.'s work (32) highlighted the balancing act that women engaged in as they struggled to manage secondary work, family commitments, coaches' training expectations and travel and playing requirements. While organizations may recognize these tensions, and in some instances family members assume caring responsibilities, the latter is not sustainable in the longer term.

With respect to *coaches*, in many leagues full-time coaches are often men with women filling part-time or second in charge positions. Research (16, 19, 38) has identified that women athletes call out the need to have access to greater numbers of women head coaches. Thus, our WASE framework highlights the important role coaches' support plays in facilitating positive gendered organizational practices. Women coaches can offer support in the relational domain that athletes value within an organization (32) and may better understand the unique challenges women athletes face. Roberts et al. (46) argue that women benefit from having strong role models, such as coaches. Research has found that women coaches often advocate and prioritize the needs of their athletes ensuring their concerns are not overlooked in a traditionally male-dominated sports organization (47). Notably, developing women and men's coaching knowledge about women's specific athletic and gender related considerations, can ensure that athletes are managed holistically and do not engage in practices that threaten long-term sustainability (19).

In complement, *teammates* can provide an important relational connection for athletes and enhance flourishing and wellbeing (19, 47). Shared aspirations can bond team members together (48), with research showing that women athletes may form a “sisterhood” to provide a culture of support, and embrace camaraderie centred around the desire to provide inspiration for other women and girls to participate in their sport (32).

The key role that the relational context of *family* provides for athlete flourishing and wellbeing cannot be under-estimated (22). The WASE incorporates consideration of families in policy and practice, through creating a family inclusive culture that celebrates connection. Non-traditional families are equally valued is a key aspect of relational support and connection. Understanding cultural and relational obligations, as well as interconnected support networks of indigenous and other cultural minorities is important for wellbeing, together cultural connection with an athlete's communities (42).

We acknowledge it is not incumbent on women athletes, coaches, teammates or family to challenge entrenched masculine practices, rather that actions and decisions that build positive cultural should be embedded within organizational practices. The proposed WASE captures the enablement and promotion of a supportive culture for women athletes, one which values intersectional identities and the uniqueness of individual experience. This recognizes women's interdependence not as isolated individuals but embedded within a supportive environment that prioritizes flourishing and wellbeing in concert with relational accountability, leading to sustainability (30). Doing so gives women athletes permission to “change the perception” of masculine sports cultures and promotes inclusive values to create a “safer open … environment” (15, p. 15). An approach that incorporates feminist flourishing enables women to exercise agency, whilst simultaneously recognizing how it can be “constrained and enabled in different ways and to different degrees among individuals in different locations in the social structure” (48, p. 470).

### Organizational practices

The WASE is underpinned by the assumption that an organization's role is to provide support mechanisms to enhance professional team athlete support and development for women in professional sports leagues. WPSTLs “bring value to the master brand [men's teams] by fostering a supportive and progressive culture that both players and fans can connect with” (16, p. 11). Research suggests that when women are valued, respected and taken seriously as athletes by an organization, they perform better on the field (49). However, women's experiences of employment are often “bolted on to the men's game, with limited consideration of their employment needs” (3, p. 7). As noted above, women's agency and voice have been constrained by conventional masculine models of professional sport. shaping the allocation of resources and athlete support practices around male athlete's need, resulting in the marginalization of women's experiences and requirements (9, 10, 49). To counter these arrangements, we propose that organizational practices to support women athletes encompass: (i) ethics of care; (ii) gender inclusive employment practices; (iii) employment contracts with a liveable salary; and (iv) revenue sharing.

Claringbould et al. (50) propose that an *ethics of care* should guide good governance and provide an environment in which people can “survive and thrive” (p. 66). Changing organizational values towards an ethic of care is a crucial aspect of undoing masculine power relations and affirming and valuing women's contribution (17). Furthermore, organizations that produce policies and practices that support women, in turn position women as legitimate and equal stakeholders in an organization (10). Actioning these considerations means that employment contracts and salaries need to be ethical, life-affirming, flexible and sustainable (16).

While *employment practices* in the form of contract arrangements for women athletes are improving, many WPTSL athletes are still positioned precariously, and their gendered needs remain unsupported (10, 14, 49, 51). As we suggested in the individual component of the framework, mandatory paid parental leave, time off after the birth of a child and childcare provision, both at training and at game times should be standard employment contract inclusions (3, 52). Women are often hesitant when taking parental or carers leave, fearing stigma and career progression may be hindered (3). There is a growing acknowledgement about the need for education about these considerations to be built into contract inclusions and together with support for returning to an athlete role after childbirth (53). Building these elements into women's contracts could address longer-term stability and a provide sense of certainty (3). Short-term contracts (individual game or season based) make it difficult for athletes to plan their lives and compromise wellbeing and performance (19). Longer contracts, extending across multiple seasons or years, make it easier for women to continue the training required to perform at a high level, and supports major life decisions, such as location, housing, education and family planning. Viable and sustainable contracts include allowances for injury prevention and management, return to play mechanisms, training loads, as well as mental health support, including time away to prevent burnout (49) and access to training facilities, sports science and other performance staff (39). Collective action from gender balanced player's unions can also hold organizations to account for ensuring that women's contract needs are addressed. These shifts can also affect how power is exercised within organizations, creating alternative frameworks and norms around contracts and legitimizing women's needs and involvement (10).

*Employment contracts* are reflected in the salary women in WPTSL are paid, with low or minimum salaries used as an indicator of lack of professionalization (4). Researchers have noted that women are commonly positioned in precarious situations through these financial arrangements (e.g., 3, 13, 51, 54). Salaries are generally tied to the league's structure, for example length of season and games played and often do not recognize the broader value of these athletes' contribution to the development of the league (10). This precarity and financial uncertainty impacts wellbeing (39), and often nebulous metrics place caveats on how and when salaries increase (10). Research has advocated for an approach acknowledging that women athlete salaries should be set at a level that negates the precarity and imbalances of power, where athletes' emotional investments are not exploited nor are they required to make unsustainable sacrifices for the opportunity to play professionally (14, 51). Salaries could be structured along a progressive sliding-scale, from semi-professional through to full-time as a league matures. A liveable salary and year-round or multi-year employment, allows time and space to focus on training, without having to work in multiple jobs (16, 38).

Sports that have begun to adopt salary structures that allocate player compensation through *revenue sharing* mechanisms (10). Under this model, women athletes receive income derived from key commercial streams, including media rights, ticket sales, and corporate sponsorships. In many cases, these systems incorporate a predetermined salary cap to regulate overall expenditure and promote competitive balance across clubs (55). Moving beyond value creation such revenue traditional metrics including financial growth or game success (winning), the proposed WASE builds in considerations of rewarding the unpaid work these women have contributed and continue to invest in the leagues to ensure they are sustainable. Payments could include community engagement, media and fan engagement, promotional activities, and mentoring young players, thus bolstering important links to broader growth of the sport (56). Recognition of women players' investment in such activities places worth onto equity, care and flourishing in remuneration decisions. Therefore, the proposed WASE prompts sport organizations to financially recognise women players' contributions on- and off-field (10) provides a holistic supportive approach.

### Community connection

Our proposed WASE includes the societal domain of equity and community connection to recognize the centrality of external actors, community and social norms. The considerations in this layer are: (i) community sport participation; (ii) government support; and (iii) media narratives and coverage.

While recent successful WPSTLs have facilitated shifts in support and acknowledgment of women's sport (56), gender norms within sport are still pervasive and require constant work to negate their impact on women and on their participation (15, 57). Repositioning *sport participation* as a right afforded to all women and girls can create a ripple-effect, particularly in traditionally male-dominated sports; challenging social gender norms and norms of inclusivity (7), leading to viable talent pathways enabling grassroots participation and retention of women and girls in these sport communities. Community sport can facilitate important community linkages by providing support and catering to all its constituents, thus signifying that women and girls are valued and supported (16).

A sport ecosystem where government embraces policies, resources and programs *support* for women and girls to have the option to choose an athlete career pathway that lead to the professional level is vital (58, 59). Policies that dismantle systemic barriers and direct funding to investments which attract and retain women athletes at the professional level include support for adequate athlete infrastructure and paid employment for women at all levels of sport (60). In this context, sport forms a community approach, that builds capacity for social change (19). Although it should be noted that Posbergh (61) cautioned about the nuances of purported “protective” policies, especially those that target restrictions based on sex differences in the name of safeguarding women fairness and equity.

Community connection, support and shifts in cultural norms, have also been facilitated through WPTSL's visibility in broadcast *media* (62). The ability of an organization to garner media coverage of women's games that is “sustained, meaningful, and prominent” (63, p. 37) contributes to shifting cultural norms. Media stories highlighting professional athletes creates aspirational pathways and challenges attitudes towards women's competitions (21). Mainstream media is a critical player in the sustainability of women's sport through appealing to fans, sponsors, and the wider general public (21, 57). Intersecting professional women's sporting achievements alongside community relations, strengthens the connection (and pathway) between profession and grassroots sport (21). Social media has the potential for community building and care in women's sport (64). Pope et al (63). suggest: “new media that are woman-created or owned might highlight the value of women's sport without reference to men's sport” (p. 37). The visibility through social media, of WPTSLs and athletes within the community, has built community connection with fans. As a result, fans report a closeness and human connection with these players that many identify and connect with (15, 57). However, social media can also be a site where discrimination and abuse of women athletes is perpetuated (13), with research showing gender-based violence is also increasingly happening online (65). Collaborative approaches that bring together athletes and media to co-design policies, protections and safe online boundaries can tackle abusive gender norms in social media.

## Discussion

The proposed WASE framework provides a basis to contest the marginalization of WPTSL athletes by privileging care, flourishing and long-term wellbeing, and inclusion while concurrently valuing the unique aspects of women's professional sport. Built around the principles of sustainability, intersectionality and care, WASE is underpinned by the notion that organizations that must adapt to support women professional athletes and their diverse lives and needs. Taking a sustainable and feminist approach challenges and resists power relations linked to commercialization and external markets and addresses previously voiced concerns about the lack of an integrated approach to the developments and considerations in the professionalization of women's sport (12). At the individual level, intersectionality is foregrounded, within inclusive and equitable organizational practices enabling belonging and empowerment that are central to women's flourishing, care and wellbeing (39). The premise of the WASE framework is to value athletes as individuals and recognize the gendered context of their lives, and the mediating effects of relational connections.

The growing popularity of WPSTL places them in an increasingly stronger position to challenge how success is understood and defined (12, 51, 66). Holding onto the value of “self-organized community-oriented, bottom-up, collaborative elements” (67, p. 77), can assist with guiding the sustainability of a WPSTL through an appreciation of professional women athletes' capacity to inspire participation in grassroots sport and foster community connection. This means not only redressing the historical underinvestment in women's sport (66), but also employing measures identified in the WASE framework. Organizational practices aligned to the elements within the framework could be deployed to negate power imbalances and structural disparities that occur when sports adopt generic structures from men's leagues (12), or embed women's teams within the pre-existing men's league, to advocate for social justice issues to be addressed. Thereby, providing substantive rather than symbolic support that allows women to flourish as athletes, as flourishing has the power to shape wellbeing (30).

The WASE emphasizes and recognizes athletes as individuals with differing needs (19). In doing so it acknowledges WPSTL as value creators, growing participation pathways. for women and girls in community sport, which can build sustainability at the elite level (66). The framework reflects the complex and inter-connected considerations of the women's professional team sports landscape.

Although variations exist across sports, leagues, and national contexts, a central premise lies in the interconnectedness of the WASE dimensions. These dimensions span from broader societal considerations of community engagement to the organizational practices of governing bodies, the interface of interdependencies influencing athlete wellbeing, and the individualized actions associated with personal and sustainable care.

Collectively these dimensions shape how professionalization plays out, with changes in any one area having direct, positive or negative, implications on the others' operation and impact. That said, decisions taken at the organizational level have shown to be highly impactful in determining financial support and leadership representation thereby ingraining path-dependent gender inequalities (12). Another critical aspect with respect to organizational practices is how the women's league relates to the sports' governing body; notably to what degree the women's competition is independent of the men's, given the persistent ongoing disparities in funding and representation of women in key leadership roles (49). Notably, Taylor et al.'s (6) research on an embedded model (AFL Men/AFL Women) found that the “impact of the trio of institutional-, organizational- and individual-level pressures, processes and decisions work in conjunction to influence the overall approach of the organization to implementing the women's team into their existing structure” (p. 482). As women's leagues grow, mature and move from co-dependency to more independent standing, these leagues can shift from being fringe and marginalized stakeholders (56).

At the individual level, women athletes across the globe continue to face sexual harassment and abuse (43), a situation that WASE acknowledges in two dimensions; interdependencies and athlete wellbeing, and through individual and sustainable care. Responsibility for addressing gender-based violence and abuse should not rest on individual athletes, but rather a whole of ecosystem accountability. For example, approaches to address these issues could include making education programs for leaders, coaches, athletes and support staff working with women mandatory (65). As noted consistently in the extant literature, women athletes experience career instability, a failure of athlete protection, precarious working conditions, low wages and insufficient support for gendered support such as parental commitments (10, 49). To remedy this situation WASE provides a framework to identify areas and actions that can be taken to address the working conditions provided to women athletes.

We argue that equity and care need to be built in as principles in policy, infrastructure, funding decisions, media coverage and leadership practices thus linking ecological responsibility with gender justice. Accentuating sustainability and care means creating workplace environments free from harassment, discrimination, and gendered violence. The focus on “taking care” of women athletes aligns to the Sustainable Development Goal 5, which is dedicated to achieving gender equality and empowering women and girls. Creating actionable targets is a key method of ensuring that gender justice is an achievable goal. A checklist was developed to support knowledge translation of the WASE framework, with key domains, indicators, and examples of organizational practices associated with athlete support and development (see Table 2). It is intended to be a reflective self-assessment tool, assisting sport administrators reflect, strengthen and refine practices that support the retention of women in professional team sport.

**Table 2:** A practical implementation tool to apply the WASE framework.

Key Indicators and organisational practices	Actioned
Individual and sustainable care	
Provide parental care leave	
Activate child-care provisions	
Enable flexible individual career pathways	
Recognise cultural obligation commitments	
Accept dual career roles	
Address gendered expectations, sexism and inequalities	
Interdependence of athlete wellbeing	
Build coach relational and cultural support to players	
Stimulate relational and cultural support between teammates	
Develop a family inclusive culture	
Organizational practices	
Ensure an ethics of care that protects and nurtures player relationships	
Introduce inclusive, viable and sustainable employment contracts	
Allow for individual employment considerations	
Activate a progressive salary slide-scale	
Provide a liveable player salary	
Introduce a revenue sharing salary model	
Establish on-and-off-field player payments	
Community connection	
Map whole-of-sport talent pathways	
Build inclusive, streamlined grassroots to professional level policies, resources and programs	
Ensure visibility of women players in broadcast and social media	
Co-design with players media policies and protections	

## Conclusion

Our Women Athlete Support Ecosystem (WASE) is conceptualized as a framework to understand the interconnected resources, relationships, and practices required for women to sustain a career in professional sport leagues. We drew on the ecological-intersectional model (17, 32) and community orientation (19) to explore various levels of influence. In developing an integrated and connected framework, we discussed how women's experiences, opportunities and challenges as professional athletes were shaped through approaches to care and wellbeing, organizational practices and governance structures and community and societal connections. The WASE reflects the multi-dimensional nature of the operating environment of women's professional team sport, connecting the individual level career and working considerations to the organizational practices and more broadly through to the macro level of community.

The development of WASE was informed by considering women's gendered needs, interdependencies and capacity for co-creation (20). This allowed us to bring in feminist flourishing (30) and position women athletes positively, albeit within a context of a persistent, implied inferiority in comparison to the men's professional league in the same sport. Despite a growing commitment to gender equality in government policy and organizational governance, persistent differences between the support provided to men's and women's sport, and gendered patterns in community sport, continue to normalize the power structures of hegemonic masculinity within men's sport (7). The interdependence of the layers of institutional, organizational and individual considerations highlights the potential of each level's facility to create opportunities for change and make a difference to women's lives in professional sport. In summary, the WASE provides an integrated perspective of the dynamics at play within women's professional sport, signifying a multifaceted interaction of societal conditions, organizational values, contestation for support and resources, and individual athlete requirements, within a highly gendered sport ecosystem. The development of the WASE contributes to sport scholarship through bringing together feminist and intersectionality conceptualizations of women's professional sport to present a coherent multi-dimensional framework that connects and intertwines layers of individual, organizational and societal considerations.

The WASE provides a framework through which governing sport organizations and leagues, coaches and support staff, women athletes and government can together forge the creation of positive career pathways for girls and women, address inequities, wellbeing and gendered roadblocks, and best support women in achieving sustainable and rewarding athlete careers and leadership roles. WASE is also relevant for professional sport players' associations and international federations that are working to improve the representation of women in organizational leadership positions, enshrining better working conditions for women through collective bargaining and revenue sharing, setting international maternity regulations, developing mental health resources and creating safe spaces to report abuse. Practically, WASE can be used to develop strategies, allocate resources and address gendered considerations to best support the women's league and women athletes. At the community level, building connections can assist women and girls' grassroots participation, and talent pipeline provision can create a sustainable base of professional athletes for the future.

## Limitations and future research

The limitations of this study provide a multitude of future opportunities for research. Firstly, the development of the WASE drew on existing literature and empirically based studies. We acknowledge the extant literature is mainly from the Global North and thus will have an inherent orientation to women's sport leagues operating in those cultural contexts. Women's professional leagues vary across sports, countries, labour regulations, degree of commercialization and stages of development. Nevertheless, we argue the principles of sustainability, equity and care could be embedded in different cultural contexts and stages of development. Research by Rind and Naz (68), while not focused on professional sport, highlights women's negotiations with everyday gender norms, institutional and mobility constraints. This resistance aligns with WASE's proposal that flourishing and wellbeing depend on transforming relational and organizational conditions.

As no primary data were collected for the framework there is scope for future research to “test” the WASE with representative stakeholders across geographic, cultural and professional sport contexts. The framework includes considerations relevant for emerging, developing and maturing leagues without differentiating the controlling bodies' business models, some leagues are not-for-profit and tied to sport governing bodies, others include commercially structured standalone team franchises. The WASE is not intended to be prescriptive and should be applied as a foundation to then tailor according to the context of a specific league.

An additional limitation relates to athletes with multi-league careers, we acknowledge that some women athletes are contracted across multiple sport organizations (local club, national team, international club/league), often with variable and disconnected support environments. Grappling with how these employment types complement or challenge the wellbeing and care of athletes is an area ripe for future investigations.

In summary, the Women Athlete Support Ecosystem (WASE) is underpinned by a scholarship on gendered labor, structural precarity, and organizational power. It addresses the challenge that women athletes face in operating within men-centric leagues. WASE is a holistic, feminist intersectional framework, that challenges the systemic masculine conceptualization and operationalization of sport that has long relegated women's involvement to unsustainable conditions. The four interdependent pillars of individual care, collective wellbeing, progressive organizational practices, and authentic community connection conceptually position institutional flourishing and labor equity as structurally inseparable. As women's professional team sports continue to evolve and navigate the way forward to a positive and sustainable model, this framework provides a crucial conceptualization of considerations. We urge organizational scholars, policymakers, and sport administrators to challenge binary, exploitative labor models and actively construct an equitable, caring, regenerative future where women athletes move from working in a sport workforce with inherent systemic precarity, to an environment that enables them to thrive and flourish.
